# Using Text Messages to Bridge the Intention-Behavior Gap? A Pilot Study on the Use of Text Message Reminders to Increase Objectively Assessed Physical Activity in Daily Life

**DOI:** 10.3389/fpsyg.2012.00270

**Published:** 2012-08-02

**Authors:** Andreas R. Schwerdtfeger, Catalina Schmitz, Matthias Warken

**Affiliations:** ^1^Health Psychology Unit, Department of Psychology, Karl-Franzens-UniversityGraz, Austria; ^2^Department of Clinical Psychology, Johannes Gutenberg-UniversityMainz, Germany; ^3^IFG – Institute for Health and ManagementMainz, Germany

**Keywords:** accelerometer, daily life physical activity, intention-behavior gap, mobile phone, reactivity, sedentary lifestyle, short message service, text reminder messages

## Abstract

Sedentarism is a serious health concern in industrialized countries throughout the world. We examined whether a text message-based intervention, targeted at increasing daily levels of physical activity, would be more effective than a standard psychoeducational intervention and a control condition. Sixty-three individuals (43 women) with a mean age of 23.7 years participated in the study. They were randomly assigned to a psychoeducational standard intervention; an augmented intervention with additional short text messages sent to the mobile phones to remind participants of their action plans, and a control condition. Objectively assessed physical activity and self-efficacy were assessed pre- and post-intervention. Participants in the control condition showed a significant decline in physical activity from pre-assessment to post-assessment, whereas participants in both intervention arms exhibited a slight increase. Moreover, the augmented intervention resulted in a marginally significant increase in self-efficacy, whereas the standard intervention resulted in a significant decrease. The findings suggest that short text messages reminding individuals of their action plans are not more effective than an intervention without text messages, although there seems to be a beneficial effect on self-efficacy, which might facilitate behavior change in the long-term. Challenging aspects of the research design (e.g., reactivity of the assessment protocol) are discussed and suggestions for future research are highlighted.

## Introduction

About 80% of individuals in industrialized countries can be considered sedentary (i.e., expending less than 10% of their daily energy in the performance of moderate and high intensity physical activity; Bernstein et al., 1999). A sedentary lifestyle is a known risk factor for various diseases (e.g., cardiovascular diseases, diabetes, osteoporosis, cancer; Warburton et al., 2006). Consequently, regular physical activity is inversely related with morbidity and mortality (for meta-analyses and reviews, e.g., Blair and Brodney, 1999; Oguma et al., 2002; Löllgen et al., 2009; Samitz et al., 2011; Sattelmair et al., 2011; Woodcock et al., 2011). In sum, there is robust evidence that physical activity (i.e., bodily movement that substantially increases energy expenditure) is reliably related to better physical health and lower mortality. Importantly, drawing on previous research, exercising at least five times a week for 30 min has been recommended as a positive impact on health (e.g., United Kingdom Department of Health, 2004). However, this amount of physical activity is rarely achieved in industrialized countries and has even been considered to demotivate individuals from becoming physically active (e.g., Sallis et al., 1986; Cox et al., 2003).

Of note, a recently published study suggested that even moderate-to-low doses of physical activity may positively impact health (Wen et al., 2011). Specifically, in this prospective cohort study, over 400,000 individuals in Taiwan were tracked for an average of 8 years to predict mortality risk from (self-reported) weekly exercise. Wen and colleagues showed that exercising 15 min a day (i.e., approximately 90 min per week) resulted in a 14% reduction in all-cause mortality, suggesting that even modest doses of regular physical activity may have beneficial effects on health. Several other studies support this finding (e.g., Andersen et al., 2000; Löllgen et al., 2009; Woodcock et al., 2011). In accordance with this, the National Institutes of Health (1996) Consensus Development Panel on Physical Activity and Cardiovascular Health recommended implementing moderate bouts of physical activity for 30 min each day. This recommendation implies various daily life physical activity episodes (e.g., occupational activities, brisk walking, cycling, yard work). Thus, there is consensus among researchers that rather than persuading individuals to engage in vigorous physical activity to secure health, it might be more promising to encourage them to increase their daily amount of moderate intensity physical activity. Importantly, this recommendation relies on the assumption that moderate-to-low doses of physical activity are accompanied by rather favorable affective responses, which – in turn – should be related to better adherence, thus ultimately increasing the likelihood of engaging in more vigorous physical exercise in the future (Consensus Development Panel on Physical Activity and Cardiovascular Health, 1996; for a discussion of this assumption, see Ekkekakis et al., 2008).

Given the robust evidence of physical and psychological benefits of even low-to-moderate doses of physical activity, various intervention studies have been carried out to examine the effectiveness of programs to reduce sedentary behavior and to increase physical activity in adults (for meta-analyses and reviews, see, e.g., Kahn et al., 2002; Foster et al., 2005; Conn et al., 2011; Williams and French, 2011). In general, it seems that intervention programs are modestly effective in reaching this aim with effect sizes ranging from 0.19 to 0.28, thus indicating rather small effects.

A possible explanation for these rather marginal effects is that interventions targeting behavior change might lose impact once participants are involved with their daily routines, which could distract them from their action plans. Weinstein (1988), for example, used a “messy desk”-analogy to illustrate the obstacles that may emerge when aiming to transform intentions into action. He argued that various factors could intervene between intentions and action. Specifically, daily routines and other competing plans struggle for attentional resources and could therefore interfere with the motivation to change behavior. To counter this, Weinstein suggested using reminders to keep intentions active. Indeed, there is evidence that periodic prompts and reminders could increase the effectiveness of health intervention programs, although analyses of long-term effects are largely missing to date (for reviews, e.g., Marcus et al., 1998; Fry and Neff, 2009).

In the last decades, new mobile technologies have become available that might prove useful to keep intentions active and, thus, to facilitate behavior change (e.g., Riley et al., 2011). In particular, mobile phones are now common companions in everyday-life, and Short Message Service (SMS) pervades the general public to nearly 100% (Patrick et al., 2008). SMS has the potential to reach an individual at any time, place, or setting, thus constituting a promising addendum of health promotion programs (for a review, e.g., Fjeldsoe et al., 2009). In a recent study, Prestwich et al. (2010) randomly assigned participants to one of three conditions. In one group, participants took part in a short psychological intervention to increase their daily amount of brisk walking by forming implementation intentions (e.g., Gollwitzer, 1993). In particular, they were instructed “to think when and where would be the most convenient or enjoyable for them to walk 30 min per day for 5 days a week in bouts of at least 10 min” (Prestwich et al., 2010, pp. 42–43). In addition, for four consecutive weeks they received text messages to remind them of their action plans (i.e., in which situation they intended to walk briskly). In a second group, participants also took part in a psychological intervention and also received text messages. However, in this group the text messages aimed to remind them of their goals (i.e., to walk briskly five times a week). Participants in the control group did not form implementation intentions but were merely informed about the governmental guidelines for physical activity and the health benefits thereof. At the beginning and at the end of the study participants completed a self-report measure of brisk walking, which constituted the main outcome measure. The results showed that participants in both intervention arms significantly increased brisk walking relative to the control group. Moreover, both intervention groups were better able to recall their plans and goals, respectively, following the intervention. However, the main outcome variable was assessed via self-report, and the study could not answer the important question whether an augmented psychological intervention (i.e., with additional text messages) is, *per se*, superior relative to a standard intervention (i.e., implementation intentions without SMS reminders), thus demonstrating incremental utility.

Hence, we were interested to examine whether the use of text message reminders in addition to a standard intervention relates to a higher amount of physical activity performed in everyday-life as compared to a standard intervention and a control group. We expected that reminding participants of their intentions to become more physically active would result in a more successful behavior change as compared to a standard intervention. Importantly, contrary to previous studies, we aimed to assess physical activity by means of objective data. That is, we recorded bodily movements by means of accelerometers the week prior to the intervention and the week following the intervention (i.e., for two consecutive weeks). Finally, we also assessed self-reported change in physical activity, self-efficacy, and participants’ satisfaction with both intervention arms.

## Materials and Methods

### Participants

Overall, 63 individuals with an age range from 18 to 34 years voluntarily agreed to participate in the study. They were recruited via oral communication and flyers distributed at the university campus. Participants were randomly assigned to one of three intervention arms: no intervention (*n* = 21, 17 women), standard psychoeducational intervention (*n* = 20, 12 women), and augmented intervention (*n* = 22, 14 women). The study was advertised as a study on objectively assessed physical activity as performed in everyday-life. Only individuals who owned a mobile phone and reported to not exercise extensively on a regular basis (i.e., exercising a maximum of 1 day a week for less than 1 h) were eligible for study participation.

### Intervention arms

The standard intervention was a solitary session (approximately 35 min) aimed at encouraging participants to increase their daily physical activity by providing information about the psychological and physical benefits of even mild doses of daily physical activity. Specifically, the intervention was grounded on to two prominent theories in health psychology, the social-cognitive theory (Bandura, 1986), and the theory of implementation intentions (Gollwitzer, 1999). In particular, participants were taught that life conditions have changed dramatically during the last centuries, and in modern societies physical activity has dramatically decreased. They were then informed about the physical and psychological short-term consequences of physical activity and that even small doses of everyday physical activity are beneficial to health (outcome expectancies). Finally, participants received information about various ways to increase daily life physical activity (e.g., using the stairs instead of the elevator, walking/cycling instead of taking the car/bus, get off the bus one stop ahead). They should then indicate whether they could carry out this activity several times a day, once every day, several times a week, once a week, or less often. Moreover, they were encouraged to think of other alternative plans of how to increase their daily amount of physical activity. In a final step, participants should quote in detail which behavior they intended to perform in which situation during the next week (when, where, and how; i.e., forming implementation intentions). Participants took part in this intervention in small groups of 4–11 individuals. The sessions were led by the second (Catalina Schmitz) and third author (Matthias Warken).

In the augmented intervention arm, participants attended the same session but additionally received short text messages on their mobile phones in the week following the session, which aimed to remind them about their action plans. Each day, only one message was sent in a time frame from 9 a.m. to 7 p.m., thus totaling seven messages throughout the week. The messages aimed to remind participants about their intentions, were formulated in variants, but were not tailored to the individual (e.g., “Do you still think of your intention to become more physically active?”, “Did you think of your intentions yet?”, “This is just to remind you of your intention to become physically active.” “Do you still know the wording of your intentions?”). Each day a different text was sent to secure attention. Participants were not requested to respond to these reminders, hence we could not verify if messages had been read. However, *post hoc* evaluations suggested that the messages were read. Text messages were sent automatically by an online service (www.sms-one.de). We refer to this group as the intervention plus SMS-group or augmented intervention group. Individuals in the control group did not receive any intervention but underwent repeated assessment of physical activity.

### Physical activity

Physical activity was recorded by means of uniaxial accelerometers (Actigraph GT1M) attached to the ankle of the non-dominant foot 1 week prior to the intervention session (week 1) and 1 week following the session (week 2). It should be noted that some research suggests that accelerometers should be attached to the hip to quantify metabolically relevant whole-body movements with sufficient accuracy (for a review, see Trost et al., 2005). However, it seems that recordings at the ankle are more sensitive over a wide intensity range of physical activity as compared to recordings at the hip, suggesting that ankle recordings might be more appropriate to index human movement (Guinhouya et al., 2005). The GT1M is well-validated and has been shown to measure physical activity with sufficient reliability (e.g., Matthews et al., 2002; Trost et al., 2005). The sensitivity of the device ranges from approximately 0.05–2.00 G (gravitation) and the relevant measure is counts/min. Activity counts were sampled at 30 Hz and stored in memory for each minute.

Moreover, ratings of perceived change in physical activity were assessed. At the end of the intervention, participants were instructed to rate on a 3-point scale to what extent they believed their physical activity had changed from pre-assessment to post-assessment (physical activity increased, stayed about the same, decreased). We decided to assess subjective change in physical activity instead of absolute activity for each week because we believe that it is easier for individuals to report relative change of a certain behavior across a period of 2 weeks as compared to absolute levels. Moreover, our aim was to validate the changes in objectively assessed physical activity with subjective ratings. Therefore, we opted for a simple change score with adequate face validity.

### Psychological and demographic measures

Use of the mobile phone was assessed via two items: How often do you use your mobile phone? How often do you carry your mobile phone with you? Answers were given on a 3-point frequency scale (seldom, sometimes, often). Familiarity with SMS was assessed via two questions: How many text messages do you send each month? How many text messages do you receive each month? Answers were given on 4-point frequency scales (between 0 and 10, more than 10, more than 50, more than 100). Responses were summed for both mobile phone use and familiarity with text messaging. Both scales were positively interrelated (*r* = 0.49, *p *< 0.001). These variables were collected to assure comparability across experimental groups.

Self-efficacy was assessed in both intervention groups immediately after the intervention session and 1 week later after the second assessment of physical activity by means of a self-constructed scale. We used a modified version of the self-efficacy scale for physical exercise (Fuchs and Schwarzer, 1994). This instrument comprises six items assessing self-efficacy with respect to the planned physical activities participants gathered in the intervention session despite of psychological, social, or contextual obstacles (e.g., “I am confident that I manage to follow my plans with respect to physical activity even when I feel tired,” “… even when I feel stressed,” “… even when I am in a hurry,” “… even when I am busy,” “… even when I am socially involved,” “… even when being physically active seems incompatible with other people’s behavior”). Individuals are instructed to rate their confidence to be physically active on a 7-point Likert scale between the poles 1 (not at all confident) and 7 (absolutely confident). The reliability of this scale was fair (Cronbach’s alpha = 0.68). This scale was not filled out by participants in the control condition because it was explicitly framed to the action plans that were developed during the intervention sessions and participants in the control condition were not asked to develop any plans to change behavior.

Furthermore, participants’ satisfaction with both intervention arms was analyzed. Therefore, they completed a short questionnaire at the end of the study evaluating whether the standard intervention was perceived as meaningful (vs. meaningless), informative (vs. pointless), helpful (vs. needless), effective (vs. ineffective), and interesting (vs. boring) on 7-point bipolar items (ranging from 1 to 7). The mean sum score of this scale was 15.17 (SD = 5.38), suggesting mediate scores of satisfaction. Internal consistency of this scale was 0.57 (Cronbach’s alpha). Although the reliability was rather low, we decided to include this measure in further analysis, because it may help exploring how the intervention session was perceived and whether satisfaction with the intervention needs to be considered when evaluating the effectiveness of these intervention programs. In order to evaluate the quality of the text messages participants in the augmented intervention group additionally filled out a short scale asking whether the text messages were perceived as meaningful (vs. meaningless), bothersome (vs. unobtrusive; reversely coded), helpful (vs. needless), joyful (vs. stressful), surprising (vs. predictable), attention grabbing (vs. unspectacular), and interesting (vs. boring) on 7-point bipolar items (ranging from 1 to 7). The mean sum score of this scale was 28.71 (SD = 7.75), suggesting that participants had a rather favorable attitude toward the messages. Internal consistency of this scale was good (Cronbach’s alpha = 0.80).

Body Mass Index (BMI) was assessed objectively by measuring participants’ weight and height. Moreover, participants worked on questionnaires assessing demographic and lifestyle variables (age, sex, smoking status). Outside temperature was assessed for each day during data collection via an online weather information system.

### Procedure

The study protocol was approved by the institutional ethics committee. Participants were enrolled in two waves. About 40% of the participants took part in the study from January to March with an average outside temperature of 2.17°C (SD = 1.40), whereas 60% of the sample took part during the months May to August with a mean outside temperature of 21.08°C (SD = 1.53). Only participants who owned a mobile phone were eligible for study participation. They were requested to report their mobile phone numbers prior to participation. Upon arrival at the department, informed consent was obtained and participants were informed that they could discontinue participation in this study any time without giving a reason. Then they were made familiar with the study protocol. They were equipped with the accelerometers and instructed to wear the devices for two consecutive weeks. Moreover, they filled out questionnaires on demographic and lifestyle variables and on their habitual use of the mobile phone. They were then randomly assigned to one of the three groups. Participants in the control group were requested to wear the devices for two consecutive weeks without taking part in an intervention. Participants in the standard intervention and augmented intervention groups also wore the accelerometers for two consecutive weeks but returned to the department after the first week to take part in a psychoeducational session of approximately 35 min duration. Participants of both intervention arms attended the psychoeducational session in mixed groups and were not informed beforehand about their membership in one of the two intervention groups. However, they were told that some of them would receive short text messages during the next week. At the end of the session, they were asked to fill out a questionnaire on self-efficacy. Individuals in the augmented intervention group received short text messages on their mobile phones the week following the intervention session in a time frame from 9 a.m. to 7 p.m. After the second assessment, participants returned to the laboratory to hand over the equipment and to fill out the self-efficacy scale and a short questionnaire on satisfaction with the intervention (only individuals in the intervention groups) and satisfaction with the text messages (only individuals in the augmented intervention group). Then their height and weight were assessed by the experimenter. We decided to measure these variables at the end of the study because of two reasons: First, we wanted to make sure that BMI-measurement could not affect the level of physical activity, because participants might change behavior in accordance with presumed expectations (i.e., being overweight could suggest lower levels of physical activity). Second, we did not expect weight to change substantially across the 2 weeks of study participation, because the intervention focused on rather low-intensity everyday-life physical activity that might not result in significant weight loss. There was no monetary compensation for study participation but participants could receive course credit when applicable and were offered a printout of their activity levels at the end of the study.

### Data parameterization and analysis

Accelerometer data were analyzed for each day between 9 a.m. and 10 p.m. We evaluated wearing time by instructing participants to record time intervals when devices were not in use and deleting these time slots. Moreover, when activity counts revealed no movement at all for at least 1 h data were deleted. Night time data were also not analyzed. Thus, the maximum wearing time summed up over 14 days could total 180 h for each individual. Across the sample, 2.20% of the cumulated recording hours were lost due to non-compliance (excluding night time). Activity counts were then aggregated for each week to yield an average score per minute. One individual in the augmented intervention group did not wear the device at all at post-assessment, thus leaving a total sample size of 21 individuals in this group.

## Results

In a first set of analyses, we aimed to examine whether the sample was sedentary (i.e., spending less than 10% of the daily energy in the performance of moderate and high intensity physical activity; Bernstein et al., 1999) by calculating the proportion of physical activity episodes (as assessed via accelerometers) prior to the intervention that were of moderate, high, or very high intensity. To quantify these intensity ranges, we used the cutoff-scores published by Matthews (2005). We found that on average 11% of activity episodes were conducted in the moderate, high, or very high intensity range, thus suggesting that the sample was indeed predominantly sedentary. Only individuals with access to a mobile phone were eligible for study participation. All participants were regular mobile phone users and were well experienced with the use of text messages. Descriptive data are presented in Table 1.

**Table 1:** **Sample characteristics and descriptive statistics**.

	Control		Intervention		Intervention + SMS		*p*
	*M*	SD	*M*	SD	*M*	SD	
Age	23.62	3.60	23.60	4.31	23.90	4.12	0.96^a^
BMI	24.12	4.23	23.86	5.15	23.09	4.82	0.77^a^
Mean outside temperature (°C)	13.10	9.84	13.34	9.69	13.86	9.41	0.97^a^

	**Percent**		**Percent**		**Percent**		

Smokers	48		25		19		0.11^b^
Female sex	81		60		67		0.33^b^

	**Mean rank**		**Mean rank**		**Mean rank**		

Use of mobile phones	30.36		31.15		32.98		0.88^c^
Familiarity with text messaging	30.79		31.58		30.65		0.98^c^

*^a^Univariate ANOVA, ^b^Cramers V, ^c^Kruskall–Wallis test*.

The mean age of the sample was 23.71 years, the mean BMI was 23.69. There were 19 smokers (31%). We analyzed whether the three experimental groups were comparable on a number of measures. Therefore, we calculated several analyses (Univariate ANOVAs, Cramers V, and Kruskal–Wallis tests) to examine possible differences in age, sex, BMI, mean outside temperature, smoking, and use of the mobile phone. The results are presented in Table 1. Overall, there were no significant differences between groups in neither variable, thus suggesting that they were comparable.

We also analyzed whether participants differed with respect to data collection wave (colder months vs. warmer months). In short, we found no significant differences with respect to age [*t*(60) = −0.21, n.s.], BMI [*t*(60) = −1.01; n.s.], sex (χ^2^ = 0.57; n.s.), smoking status (χ^2^ = 0.14, n.s.), use of mobile phones (Mann–Whitney-*U* = 351.50, n.s.), and familiarity with text messages (Mann–Whitney-*U* = 396.50, n.s.), thus suggesting that participants were comparable across data collection waves.

**Figure 1:**
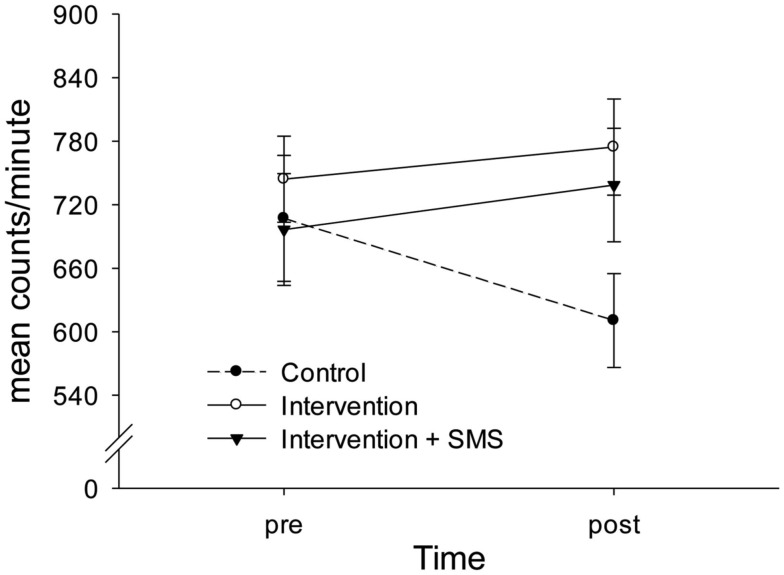
**Interaction of group and time on objectively assessed physical activity**. The control group showed a significant decline in physical activity from pre- (week 1) to post-assessment (week 2), whereas both intervention groups increased their levels of physical activity moderately. There was no significant difference between the standard intervention and the augmented intervention group (with text messages). Whiskers indicate ± 1 standard error.

Next, we analyzed if there was a difference in mean physical activity between the three groups from pre-assessment to post-assessment. Therefore, we calculated a repeated measures-ANOVA with group as between-subject factor (control, intervention, intervention plus SMS) and time as within-subject factor (pre- vs. post-assessment). There was a significant interaction of group and time [*F*(1, 59) = 4.07, Wilks-λ = 0.88, *p* = 0.02, η_p_^2^ = 0.12], indicating that groups differed with respect to the time course of physical activity. *Post hoc* *t*-tests suggested that groups did not differ on pre-assessment (control: *M* = 707.15, SD = 273.01; intervention: *M* = 744.15, SD = 202.99; intervention plus SMS: *M* = 696.52, SD = 242.42). However, at post-assessment there was a significant difference between the control group (*M* = 610.57, SD = 203.57) and the standard intervention group [*M* = 774.49, SD = 202.99; *t*(39) = −2.58, *p* = 0.01, Cohen’s *d* = 0.81], and a marginally significant difference between the control group and the augmented intervention group [*M* = 738.62, SD = 245.69; *t*(40) = −1.84, *p* = 0.07, Cohen’s *d* = 0.57)]. Further analyses revealed that only the control group showed a significant change (i.e., decline) from pre- to post-assessment [*t*(20) = 2.72, *p* = 0.01, Cohen’s *d* = −0.41)]. Physical activity in both intervention arms did not change significantly [intervention: *t*(19) = −0.77, *p* = 0.45, Cohen’s *d* = 0.16; augmented intervention: *t*(20) = −1.06, *p* = 0.30, Cohen’s *d* = 0.17]. This pattern of result is depicted in Figure 1.

To examine whether groups differed in subjectively reported change in physical activity, we analyzed participants’ reports of whether they believed their physical activity level had changed from pre-assessment to post-assessment. Overall, 27% believed that their daily amount of physical activity had increased, 63% believed that it stayed about the same, and 10% were of the opinion that it had actually decreased. There was a significant correlation between the subjectively reported change in physical activity and the objectively assessed pre-/post-difference (Spearman rank ρ = 0.44, *p *< 0.001). Hence, both subjectively reported and objectively obtained changes in physical activity were moderately interrelated, thus confirming validity of the assessment. Furthermore, we calculated a non-parametric Kruskal–Wallis test with group (control, intervention, augmented intervention) as between-subject factor and subjectively reported change in physical activity as an ordinal dependent variable. There was a significant effect for group (Kruskal–Wallis *H* = 12.38, *p* = 0.002), indicating that individuals in the control group more frequently reported a decline in physical activity (29% of the participants) as compared to the intervention group (0%) and the augmented intervention group (0%). On the contrary, 53% of the intervention group believed that they increased their activity level from pre-assessment to post-assessment as compared to 35% in the augmented intervention group, and 12% in the control group. Thus, the subjective ratings complement the objectively obtained result that a substantial proportion of individuals in the control group showed a decline in physical activity from pre-assessment to post-assessment, whereas individuals in the intervention groups more frequently showed an increase.

**Figure 2:**
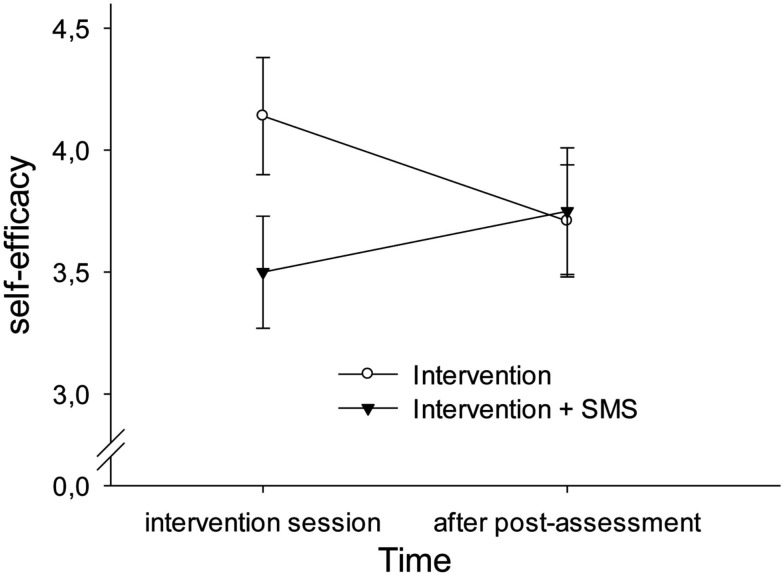
**Interaction of group and time on self-efficacy**. The augmented intervention group (with text messages) showed an increase in self-efficacy ratings after post-assessment (week 2), whereas the standard intervention group showed a decline. However, both groups marginally differed immediately after the intervention session with the augmented intervention group showing lower ratings than the standard intervention group. Whiskers indicate ± 1 standard error.

Moreover, we examined whether the intervention groups differed significantly in self-efficacy. Therefore, we calculated a repeated measures-ANOVA with group as between-subject factor (intervention vs. intervention plus SMS) and time as within-subject factor (intervention session vs. after post-assessment). We found a significant interaction of group and time [*F*(1, 37) = 12.15, Wilks-λ = 0.75, *p* = 0.001, η_p_^2^ = 0.25]. This effect was due to a significant decline in self-efficacy in the intervention group [*t*(17) = 3.44, *p* = 0.003, Cohen’s *d* = 0.40], whereas there was a tendency for the augmented intervention group to show an increase [*t*(20) = −1.72, *p* = 0.10, Cohen’s *d* = 0.22]. Moreover, there was a marginally significant difference between groups after the intervention session but no significant difference after post-assessment [*t*(39) = −0.01, *p* = 0.99]. In particular, the intervention group showed higher self-efficacy ratings after the intervention session (*M* = 4.14, SD = 1.02) as compared to the augmented intervention group [*M* = 3.50, SD = 1.06, *t*(37) = 1.91, *p* = 0.07, Cohen’s *d* = 0.61]. Figure 2 depicts the Group × Time interaction for self-efficacy.

We also calculated Pearson correlations to analyze the relationship between satisfaction with the intervention session and the pre-difference/post-difference in physical activity (activity post-intervention − activity pre-intervention). The correlation was *r* = 0.32 (*p *< 0.05), indicating that those individuals who were more satisfied with the intervention showed a stronger increase in objectively assessed physical activity from pre-assessment to post-assessment.

Finally, we exploratively tested whether the outside temperature had an impact on the effectiveness of the intervention. Therefore, we re-calculated the repeated measures-ANOVA with group and wave as between-subject factors, and time as within-subject factor. There was a main effect for wave [*F*(1, 56) = 8.60, *p* = 0.005, η_p_^2^ = 0.13], indicating that participants were physically more active during warmer temperatures (*M* = 773.65, SD = 211.02) as compared to colder temperatures (*M* = 618.66, SD = 177.01; Cohen’s *d* = 0.80). Moreover, we could confirm the Group × Time interaction [*F*(2, 56) = 3.29, Wilks-λ = 0.90, *p* = 0.045, η_p_^2^ = 0.11], thus replicating that participants in the control group showed a decline of physical activity from pre-assessment to post-assessment, whereas participants in both intervention arms showed a non-significant increase. Of note, there was no significant Group × Wave × Time interaction [*F*(2, 56) = 1.17, Wilks-λ = 0.96, *p* = 0.32], thus indicating that outside temperature did not differentially impact the effectiveness of the interventions.

## Discussion

The study’s aim was to examine whether an augmented intervention (with occasional SMS reminders of formerly expressed intentions) would be more effective in increasing objectively assessed physical activity relative to a standard intervention and a control condition. We found a significant interaction of experimental group and time (pre-intervention vs. post-intervention), documenting that individuals in the control group showed decreasing activity levels from pre-assessment to post-assessment. This effect was moderate in size (*d* = −0.41). In contrast, individuals in both intervention arms slightly – however not significantly – increased their physical activity levels as became evident by rather small effect sizes (*d* = 0.16 and *d* = 0.17, respectively). Of note, contrary to expectation, individuals in the augmented intervention arm did not increase activity above those in the standard intervention arm. This finding suggests that an augmented intervention with additional short text reminders is not more effective in changing behavior as compared to a standard psychoeducational intervention, at least not in the short run. It should be noted, however, that we did not control whether text messages had been actually read by the participants or had been discarded without grabbing attention. Hence, the failure to show a beneficial effect of the augmented intervention might be explained by non-adherence with the instructions. However, as the evaluation of the text messages revealed, participants were rather positive about the messages, thus suggesting that the messages had been processed to a certain degree. Nonetheless, in order to secure adherence in future studies participants could be requested to reply to these messages.

Of note, findings for subjectively reported change in physical activity confirmed the general pattern of result. That is, individuals in the control group acknowledged to a greater extent that they exhibited a decline in physical activity during post-assessment as compared to pre-assessment, whereas individuals in both intervention arms were to a larger degree of the opinion that they increased their daily amount of physical activity.

It is particularly striking that participants in the control group showed a substantial decline in physical activity from pre-assessment to post-assessment. This shrinkage might be interpreted in terms of the reactivity of the assessment protocol. Specifically, when participants are aware that physical activity is assessed by means of accelerometers they may increase their daily level of physical activity just to put the devices to a test. Indeed, it has been discussed that methodological reactivity is a challenging topic in ambulatory monitoring studies in general (e.g., Fahrenberg, 1996; Barta et al., 2012). Notably, a recent study seems to support our finding of a tentative reduction in objectively assessed physical activity from pre- to post-intervention. Using a similar research design Motl et al. (2012) found that patients with multiple sclerosis undergoing a social-cognitive internet intervention to increase physical activity showed an approximately 30% decline in accelerometer counts in the week following the intervention. Interestingly, pedometers are powerful tools to increase daily amounts of physical activity by instructing individuals to reach a certain goal each day (e.g., 10,000 steps per day; Kang et al., 2009). Although, in our study the daily amount of physical activity could not be retrieved by the participants, the accelerometers may have tempted them to become physically more active because the procedure was novel. Conversely, when individuals were familiarized with the devices (on post-assessment), they seemed to adjust their daily amount of physical activity to normal (i.e., sedentary) levels. In accordance with this interpretation, the average percentage of moderate, high, or very high intensity physical activity dropped from 11% at pre-assessment to 9% at post-assessment. Hence, although the psychoeducational interventions did not result in reliable increases of physical activity, they seemed to have prevented a decline of physical activity to sedentary levels, which was observed in the control group, indicating reactivity of the measure.

Of note, the standard intervention group showed a significant decrease in self-efficacy (*d* = −0.40), whereas individuals in the augmented intervention arm exhibited a moderate increase in self-efficacy (*d* = 0.22). This finding may suggest that individuals in the standard intervention group became discouraged by the experience that they could not fully transform their intentions into action. On the contrary, the text message reminders in the augmented intervention group might have assisted sedentary individuals to strengthen the confidence to become more active. Importantly, there is robust evidence that self-efficacy is a prerequisite for successful behavior change throughout a variety of health-related domains, including physical activity (e.g., Holden, 1991; Rovniak et al., 2002; Sharma and Sargent, 2005; Gwaltney et al., 2009). Moreover, several recent studies have shown that self-efficacy mediates the effects of health promotion interventions on objectively assessed physical activity (e.g., Burke et al., 2008; Dutton et al., 2009; Darker et al., 2010). Thus, our results tentatively suggest that, although the augmented intervention was not related with elevated levels of physical activity, it might have increased the likelihood of the participants to become more active in the long run, because they were more confident to do so. We have to admit, however, that the standard intervention group showed a tendency toward higher self-efficacy ratings as compared to the augmented intervention group after the intervention session. This difference was moderate in size and may suggest that both groups showed a regression to the mean at post-assessment, which could have biased the finding. Hence, further studies are needed to verify the psychological benefits of text message reminders for increasing daily life physical activity.

Notably, although outside temperature was meaningfully related to the amount of physical activity exhibited in daily life (i.e., during warmer months there was more activity than during colder months), this variable did not interact with experimental group, documenting that both interventions prevented a decline in physical activity in the course of the study irrespective of weather conditions.

Taken together, the findings of this pilot study suggest that psychoeducational interventions are moderately successful in facilitating physical activity in the short-term. There was, however, no evidence of a beneficial effect of short text message reminders to keep intentions active during post-assessment. In our view, there are several reasons for this. In the following, we will discuss these reasons and offer recommendations for future research:

1. When examining the impact of an intervention on objective physical activity, researchers are advised to familiarize participants with accelerometers prior to assessing baseline activity in order to account for the reactivity of the method. In this study, individuals exhibited comparably high levels of physical activity prior to the intervention, thus overestimating baseline physical activity in everyday-life. Consequently, the change from pre-assessment to post-assessment in both intervention groups was most likely underestimated.
2. Although the intervention was generally perceived well, there were individuals who were not satisfied and those individuals did obviously not benefit from the intervention. Given that satisfaction with the intervention was positively correlated with the amount of physical activity change from pre-assessment to post-assessment, future research should identify individuals who are most likely to benefit from this kind of intervention and those who might not.
3. Text messages were sent out once a day (randomly from 9 a.m. and 7 p.m.) throughout the week following the intervention. Thus, the time interval between the intervention session and the mobile reminders might have been too short to effectively contrast this condition with the standard intervention arm. In keeping with Weinstein’s “messy desk”-analogy, we would recommend sending out reminders with a longer latency, i.e., when the impact of the intervention session is likely to fade out (e.g., 3–4 weeks after the intervention).
4. Text messages were provided in a non-tailored fashion. That is, participants received standard messages asking if they still thought of their intentions. Future research might want to tailor messages more closely to the personal needs of the individual (with respect to timing and content), because previous research has shown that tailoring messages to the needs of the particular individual is more effective than a “one size fits all”-approach (e.g., Noar et al., 2007; Latimer et al., 2010).

Nonetheless, our findings suggest that a single psychoeducational intervention might be effective in increasing everyday-life physical activity in the short run. Moreover, short text messages reminding participants of their action plans compiled during the intervention session could be an effective tool for increasing self-efficacy to become physically active, which is a well-known antecedent of successful behavior change.

## Conflict of Interest Statement

The authors declare that the research was conducted in the absence of any commercial or financial relationships that could be construed as a potential conflict of interest.
